# The Antimicrobial Peptide Human Beta-Defensin 2 Inhibits Biofilm Production of *Pseudomonas aeruginosa* Without Compromising Metabolic Activity

**DOI:** 10.3389/fimmu.2020.00805

**Published:** 2020-05-08

**Authors:** Kevin R. Parducho, Brent Beadell, Tiffany K. Ybarra, Mabel Bush, Erick Escalera, Aldo T. Trejos, Andy Chieng, Marlon Mendez, Chance Anderson, Hyunsook Park, Yixian Wang, Wuyuan Lu, Edith Porter

**Affiliations:** ^1^Department of Biological Sciences, California State University, Los Angeles, Los Angeles, CA, United States; ^2^Department of Chemistry and Biochemistry, California State University, Los Angeles, Los Angeles, CA, United States; ^3^Institute of Human Virology and Department of Biochemistry and Molecular Biology, University of Maryland School of Medicine, Baltimore, MD, United States

**Keywords:** airways, antimicrobial peptides, biofilm, cystic fibrosis, epithelial cells, innate immunity, mucosa, *Pseudomonas aeruginosa*

## Abstract

Biofilm production is a key virulence factor that facilitates bacterial colonization on host surfaces and is regulated by complex pathways, including quorum sensing, that also control pigment production, among others. To limit colonization, epithelial cells, as part of the first line of defense, utilize a variety of antimicrobial peptides (AMPs) including defensins. Pore formation is the best investigated mechanism for the bactericidal activity of AMPs. Considering the induction of human beta-defensin 2 (HBD2) secretion to the epithelial surface in response to bacteria and the importance of biofilm in microbial infection, we hypothesized that HBD2 has biofilm inhibitory activity. We assessed the viability and biofilm formation of a pyorubin-producing *Pseudomonas aeruginosa* strain in the presence and absence of HBD2 in comparison to the highly bactericidal HBD3. At nanomolar concentrations, HBD2 – independent of its chiral state – significantly reduced biofilm formation but not metabolic activity, unlike HBD3, which reduced biofilm and metabolic activity to the same degree. A similar discrepancy between biofilm inhibition and maintenance of metabolic activity was also observed in HBD2 treated *Acinetobacter baumannii*, another Gram-negative bacterium. There was no evidence for HBD2 interference with the regulation of biofilm production. The expression of biofilm-related genes and the extracellular accumulation of pyorubin pigment, another quorum sensing controlled product, did not differ significantly between HBD2 treated and control bacteria, and *in silico* modeling did not support direct binding of HBD2 to quorum sensing molecules. However, alterations in the outer membrane protein profile accompanied by surface topology changes, documented by atomic force microscopy, was observed after HBD2 treatment. This suggests that HBD2 induces structural changes that interfere with the transport of biofilm precursors into the extracellular space. Taken together, these data support a novel mechanism of biofilm inhibition by nanomolar concentrations of HBD2 that is independent of biofilm regulatory pathways.

## Introduction

Biofilms are composed of microbial communities encased in a protective layer of self-produced, extracellular polymers. Biofilms are formed on both abiotic and biotic surfaces and play a significant role in a variety of settings such as aquaculture (1), the food industry, and the clinical field as a factor for antimicrobial drug resistance. Biofilms can colonize body surfaces and mechanisms regarding how our bodies prevent biofilm formation are under extensive investigation (2). In part, biofilms provide tolerance to host immune factors and antibiotics through impeding their diffusion. Furthermore, biofilms enhance bacterial resistance to these factors by altering bacterial metabolism resulting from the decreased oxygen levels in the center of the biofilm mass as well as the acidification of the local microenvironment (2–5). The biofilm matrix is primarily composed of exopolysaccharide, proteins, and extracellular DNA and has been particularly well studied in *Pseudomonas aeruginosa*, a ubiquitous, opportunistic, Gram-negative bacterium. The major structural polysaccharides of *P. aeruginosa* biofilms are Pel, which is composed of positively charged amino sugars, and Psl, which is a polymer of glucose, rhamnose, and mannose; and in certain strains, alginate – an anionic polysaccharide (6–8). Proteinaceous components of biofilm include type 4 pili and cup fimbriae serving attachment and various proteins that connect matrix components adding strength to the biofilm (9). Extracellular DNA (eDNA), which is released via cell lysis (10), plays an important role in priming surfaces for the initial adhesion of the bacteria as well as in maintaining the structural integrity of the polysaccharide fibers (3, 6, 11–14).

Multiple regulatory networks govern the complex process of biofilm formation (15), which progresses from initial attachment mediated by the flagella and the production of pili, to downregulation of flagellar genes, upregulation of the production and secretion of matrix components, maturation, and eventual reappearance of flagella and dispersion. For *P. aeruginosa*, biofilm regulation has been well studied and several regulatory systems have been identified including the Las, Rhl, and quinolone quorum sensing systems, the GacA/GacS two-component system, and c-di-GMP controlled pathways. Key quorum sensing molecules for Las, Rhl, and quinolone systems are N-(3-oxododecanoyl)-homoserine lactone (3-oxo-C12-HSL), N-butanoyl-homoserine lactone (C4-HSL), and 2-heptyl-3-hydroxy-4-quinolone (known as Pseudomonas Quorum Sensing molecule or PQS), respectively (16, 17). These overlapping regulatory systems not only control the production of biofilm but also the production of pigment and various other virulence factors (17, 18). Genes whose expression is modulated during biofilm formation include *flgF*, which encodes for the basal rod in bacterial flagellin, and *pslA*, which is the first gene in the polysaccharide synthesis locus (19, 20).

In addition to being able to produce biofilm, *P. aeruginosa* possesses potent virulence factors such as: a type III secretion system, which allows it to directly deliver exotoxins to host cells (21); rhamnolipids, which enable *P. aeruginosa* to disrupt the tight junctions of respiratory epithelia (22); and pigments with diverse functions in metal-chelation, competitive inhibition of other bacteria, and resistance to oxidative stress (23–25). All of these virulence factors and resistance mechanisms contribute to *P. aeruginosa* being one of the leading isolates in healthcare-associated pneumonia in intensive care units and chronic lung infection in patients with cystic fibrosis, a genetic disorder characterized by impaired anion transport and increased mucous viscosity (26). Yet, despite its ubiquity in nature and its prevalence in healthcare-associated infections, *P. aeruginosa* is not known to cause lung infection in healthy adults, suggesting that humans possess effective innate defense mechanisms in the airways against this organism.

Antimicrobial peptides (AMPs) are small, highly conserved effector molecules that play a key role in innate immunity (27, 28). Present in plants, insects, and mammals, most AMPs are between 2 and 5 kDa in size and are cationic with varying degrees of hydrophobicity. Upon the detection of microbial components via pattern recognition receptors, AMPs can be synthesized by epithelial cells and myeloid cells as part of the first line of defense against microbes (29–33). A wealth of research has been performed on the ability of AMPs to displace cations bound to bacterial membranes, which are rich in either negatively charged lipopolysaccharides or lipoteichoic acids in addition to anionic phospholipids (34). After binding to bacterial membranes, AMPs can perturb the membrane structure and form pores mediated by hydrophobic and electrostatic forces. In addition to the charge of the membrane, phospholipid species and the presence or absence of cholesterol, which is absent in bacterial membranes, also affect the binding and orientation of AMPs and hence, their pore-forming capabilities (35–40). While pore-formation has been a widely studied mechanism of action, an increasing body of research suggests that the antimicrobial activity of AMPs may also depend on other mechanisms – disruption of cell wall synthesis, metabolic activity, ATP and nucleic acid synthesis, and amino acid uptake (33, 41). Furthermore, certain AMPs interact with the eukaryotic host cells and have immunoregulatory functions in addition to their antimicrobial activity. A notable example is that LL-37 can also: act as a chemotactic agent to recruit other immune cells and modulate cytokine and chemokine expression in host cells, bind bacterial lipopolysaccharide, and dysregulate the expression of genes involved in biofilm formation (42–46). Other AMPs have also shown multi-functional capabilities, in particular human beta-defensin 2 (HBD2) and 3 (HBD3), which have been proven to possess mechanisms of action that are more complex than simple pore formation and membrane perturbation (47–49). In fact, HBD2 was the first human beta defensin to demonstrate chemotactic activity (50). Beta-defensins are characterized by three, antiparallel β-strands stabilized by three conserved disulfide linkages preceded by an α-helical domain near the N-terminus (51–53). Although HBD2 and HBD3 share amino acid sequence and some structural similarities, their overall net charge, hydrophobicity, and charge distribution differ significantly (Table 1) and may play a role in their unique and distinct mechanisms of action. Expression of HBD2 and HBD3 is low or absent during steady state but both peptides are induced in airway epithelial tissues during infection or inflammation (31, 32, 48, 54).

**TABLE 1:** Human beta defensins-2 and -3 physicochemical properties.

**Peptide**	**Amino acid sequence^a^**	**MW (Da)**	**Net Charge**	**Hydrophobicity index**	
				**Kyte-Doolittle^b^**	**Wimley-White^c^**
HBD2	GIG*D*PVTC^1^L**K**SGAIC^2^HPVFC^3^P**RR**Y**K**QIGTC^2^GLPGT**K**C^1^C^3^**KK**P	4,328.22	6	−0.1	6.16
Linear HBD2	GIG*D*PVTAL**K**SGAIAHPVFAP**RR**Y**K**QIGTAGLPGT**K**AA**KK**P	4,141.88	6	−0.21	8.62
HBD3	GIINTLQ**K**YYC^1^**R**V**R**GG**R**C^2^AVLSC^3^LP**K***EE*QIG**K**C^2^ST**R**G**RK**C^1^C^3^**RRKK**	5,155.19	11	−0.7	12.65

*^*a*^Amino acid sequences are given in one-letter code starting from the N and ending with the C terminus. Underlined residues denote mutation sites for the linearized HBD2. Cationic residues are in boldface. Anionic residues are italicized. ^*b*^Values were calculated based on the Kyte–Doolittle hydrophobicity scale (120) using the grand average of hydropathy (GRAVY) program. Higher values represent an increase in hydrophobicity. ^*c*^Values were calculated based on the Wimley–White whole residue hydrophobicity interface scale (121) using the APD3 antimicrobial peptide calculator and predictor. Lower values represent an increase in hydrophobicity. ^1–3^ Numbers denote disulfide bond connectivity.*

Due to their lasting potency for millions of years and the feasibility of modifying AMP structures, AMPs continue to be in the spotlight as potential antimicrobial agents (33). The importance of biofilm in the infection process and in their resistance to antimicrobial agents has been recognized, yet there is a lack of drugs that interfere with biofilm. Therefore, knowledge on the structure-function relationships of AMPs, and the effects of AMPs on bacterial biofilm formation may benefit rational engineering and design of novel AMP variants and therapeutic regimens that are effective against microbial biofilms (55). Considering the induction of HBD2 and HBD3 and their secretion to the epithelial surface in response to bacteria and their products, we hypothesized that HBD2 and HBD3 have biofilm inhibitory activity. We discovered that biofilm and metabolic inhibition are proportionally reduced by HBD3 but not by HBD2. At low concentrations, HBD2 inhibits biofilm production, but not metabolic activity. We undertook multiple approaches to delineate the underlying mechanism for the selective biofilm inhibitory effects of HBD2. This research may lead to the identification of novel targets for the engineering of antimicrobials, which, in the era of increasing multi-drug resistance, is of great importance.

## Materials and Methods

### Antimicrobial Peptides

Chemical synthesis and purification of human beta-defensin 2 (HBD2/L-HBD2), its D- form (D-HBD2) comprised entirely of D-amino acids, its linearized mutant (Linear HBD2 with alanine replacing all cysteine residues), and human beta-defensin 3 (HBD3, in L-form) have been described previously (56, 57). Table 1 summarizes their physicochemical properties. Stock solutions (500 μM) were prepared in 0.01% acetic acid and stored at −20°C. For experiments, peptides were used as 10-fold concentration in 0.01% acetic acid.

### Bacterial Culture

For this study, a pyorubin-producing *P. aeruginosa* strain (a cystic fibrosis isolate previously obtained from Dr. Michael J. Welsh, University of Iowa, Iowa City) and *Acinetobacter baumannii* ATCC 19606 were used. For each experiment, snap-frozen 18 h cultures in Tryptic Soy Broth (TSB) (Oxoid) were quickly thawed, subcultured into prewarmed TSB (750 μL into 50 mL), and brought to mid-log growth phase (3 h at 37°C, 200 rpm). Bacterial cells were then sedimented and washed with 140 mM NaCl by centrifugation for 10 min at 805 × *g* in a precooled centrifuge (4°C), and resuspended in 500 μL 140 mM NaCl. For gene expression analysis, the suspended bacteria were used directly. For all other assays, the concentration of bacteria was first adjusted to 5 × 10^7^CFU/mL in 140 mM NaCl, and then further diluted as needed.

### Biofilm Quantification

In a round bottom 96-well polystyrene microtiter plate (Costar #3795), 90 μL mid-logarithmic growth phase bacteria were added to 10 μL of 10-fold concentrated defensin or 0.01% acetic acid as solvent control to yield the following final assay conditions: 1 × 10^6^ CFU/mL, 10% Mueller-Hinton broth (Oxoid, without cations), and 140 mM NaCl. Samples were incubated for 18 h at 37°C and biofilms were quantified according to Merritt et al. (58). Briefly, the content of sample wells containing non-adherent bacteria (planktonic and/or dead) was carefully discarded without disturbing the biofilm, and the well walls were rinsed three times with dH_2_O (200 μL/well) followed by addition of 125 μL of 0.1% crystal violet (Sigma-Aldrich, St. Louis, MO, United States). After 10 min incubation at RT, the crystal violet solution was removed, wells were rinsed three times with dH_2_O (200 μL/well) and air dried for at least 30 min. To solubilize crystal violet bound to biofilm, 200 μL of 30% acetic acid was added to each well and after 15 min incubation at RT 125 μL was transferred to optically clear flat-bottom 96-well polystyrene microtiter plates (Perkin Elmer from Waltham, MA, United States). Absorbance was read at 570 nm using a Victor X3 Plate Reader (Perkin Elmer). Wells containing only 125 μL of 30% acetic acid were used to subtract baseline absorbance values from samples for analysis.

### Metabolic Activity Measurement

Resazurin reduction was employed as a measure of bacterial metabolic activity (59, 60). Metabolites accumulating during bacterial growth reduce the weakly fluorescent resazurin to the highly fluorescent resorufin. Samples were prepared as described above but with resazurin (Sigma) added to the assay buffer to obtain a final concentration of 0.01% resazurin (w/v). Relative fluorescent units (RFU) were measured every 3 h with a preheated Victor X3 Plate Reader (Perkin Elmer) at 530 nm excitation and 616 nm emission wavelength and a top read.

### ATP Quantification

ATP concentrations of non-adherent bacteria were determined using the BacTiterGlo kit (Promega), with ATP standard curves prepared according to the manufacturer’s instructions. Bacteria were prepared and incubated with defensins for 18 h as described for the biofilm assay. Then, the entire well contents were transferred to a new 96 well plate, thoroughly resuspended, and of this 75 μL from each well was transferred to a black 96-well half area plate (Perkin-Elmer). After addition of 75 μL ATP substrate solution to each well and 5 min mixing on an orbital shaker, luminescence was quantified with a Victor X3 plate reader. Seventy-five μL aliquots of serially diluted ATP standard were treated in the same way.

### Pyorubin Quantification

Pyorubin is a collection of pigments produced by certain *P. aeruginosa* strains including our test strain. Although its full chemical composition is unknown, it consists of at least two, water-soluble, red-colored pigments (61). Pyorubin quantification was based on Hosseinidoust et al. (23). Briefly, bacteria were grown for 18 h in 10% Mueller-Hinton and 140 mM NaCl in the presence of 0.125–1 μM of HBD2 or solvent control in final assay volumes of 1 mL in 12-well microtiter plate (non-tissue culture treated, Costar). After 18 h incubation, well contents were collected and centrifuged at 5,000 × *g* for 10 min at 4°C to remove non-adherent bacteria. Equivolume mixtures of cell free supernatant (900 μL) and chloroform (900 μL) were mixed and centrifuged at 12,000 × *g* for 15 min at 4°C to separate the aqueous and organic phases and remove cell debris and other molecules. The aqueous phase containing pyorubin was lyophilized, dissolved in 125 μL volume of dH_2_O. From this, 100 μL were transferred to a 96-well flat bottom plate (Perkin Elmer) followed by an absorbance reading at 535 nm using a Victor X3 Plate Reader (Perkin Elmer).

### *In silico* Molecular Docking Studies

The *in silico* modeling of binding between QS molecules and HBD2 was performed using Autodock Vina (The Scripps Research Institute) through the UCSF Chimera program^1^. LasR receptor (RSCB 3IX3) and HBD2 (RSCB 1FQQ) were considered as rigid receptors and were docked with *N*-(3-oxododecanoyl) homoserine lactone (3-oxo-C12-HSL), *N*-butanoyl homoserine lactone (C4-HSL), and 2-heptyl-3-hydroxy-4-quinolone (PQS) as ligands. Phosphorylcolamine (NEtP) was used as a negative control. Free energy of binding was used to calculate dissociation constants using Eq. (1) with R = 0.00198 kcal/(mol K) and *T* = 37°C = 310.15 K (62).

(1) $$K_{D,p\,r\,e\,d}=e^{\left(\left(\left[\Delta \,G_{b\,i\,n\,d}\right]/\left[\left(R/1000\right)\times T\right]\right)\right)}$$

### Gene Expression Analysis

Mid-logarithmic growth phase bacteria were prepared and washed as described above. The assay was up-scaled using 12-well polystyrene flat bottom plates with non-reversible lids with condensation rings (Genesee Scientific, San Diego, CA, United States). Twenty μL of the washed bacteria was added to HBD2 or solvent (100 μL of 10-fold concentrated defensin in 0.01% acetic acid or 0.01% acetic acid, respectively, diluted in 900 μL 10% Mueller Hinton/140 mM NaCl) yielding about 1 × 10^8^ CFU/mL. After incubation at 37°C for the specified time points, biofilm and planktonic phase bacteria were homogenized by 10 min vortexing with 1 mm glass beads and tightly secured lids (Sigma-Aldrich, St. Louis, MO, United States). RNA extraction was performed on the homogenized samples using an RNeasy Mini Kit (Qiagen, Hilden, Germany) following the manufacturer’s enzymatic lysis and mechanical disruption protocol with acid-washed 425–600 μm glass beads (Sigma-Aldrich). Residual genomic DNA was removed with in-solution TurboDNase treatment (2 U/μL, Invitrogen, Carlsbad, CA, United States) according to the manufacturer’s recommendations followed by purification and concentration of RNA samples with RNA Clean and Concentrator-5 kit (Zymo Research, Irvin, CA, United States). Purity of RNA was confirmed by lack of amplification in SsoAdvanced^TM^ Universal SYBR^®^ Green (Bio-Rad, Hercules, CA, United States) real-time PCR using the RNA samples as template and primers for the housekeeping gene *gapA* (see Table 2). Confirmed pure RNA samples were reverse transcribed with iScript Reverse Transcription Supermix (Bio-Rad) and resulting cDNA was diluted to 25 ng/μL in nuclease free water. SsoAdvanced^TM^ Universal SYBR^®^ Green real-time PCR was performed with target primers for *pslA* and *flgF* and housekeeping gene *gapA* as reference gene (see Table 2, used at 0.75 μM final concentrations) in 10 μL reaction volumes and 12.5 ng cDNA input. Primers (Integrated DNA Technology’s, IDT, Coralville, IA, United States) were designed using IDT’s primerQuest Tool. Quantitative PCR (qPCR) and subsequent melt curve was performed using BIO-RAD’s CFX96 Real Time Thermocycler following standard conditions with annealing/extension at 60°C. CT values and relative gene expression were determined with BIO-RAD’s CFX Maestro Version 1.1. Amplified products were verified through size determination via standard agarose gel electrophoresis and melt curve analysis. Each time point was assessed in three independent experiments conducted in duplicates for a total *n* of 6. Initially, *16S rRNA* was considered as a second housekeeping gene. However, its CT values (around 5) were substantially earlier than the CT values for the target genes and *gapA* (at or above 20) and thus, *16S rRNA* gene expression was not further evaluated in this study.

**TABLE 2:** Primers used in this study.

**Gene target**		**5′**–**3′ sequence**	***T*_M_ (°C)**	**Product size (bp)**	**Product melt peak (°C)**
*pslA*	F	CGTTCTGCCTGCTGTTGTTC	56.9	160	88.5
	R	TACATGCCGCGTTTCATCCA	57.3		
*gapA*	F	CCATCGGATCGTCTCGAA	61.0	130	88.0
	R	GTTCTGGTCGTTGGTGTAG	60.0		
*flgF*	F	ACAACCTGGCGAACATCTC	62.0	137	89.0
	R	GCCATGGCTGAAATCGGTA	62.0		

### Outer Membrane Protein Profile Analysis

*Pseudomonas aeruginosa* outer membranes were harvested after incubation with HBD2 or solvent control according to Park et al. (63) with minor modifications. Briefly, bacteria were prepared as above and then grown for 18 h in 10% Mueller-Hinton and 140 mM NaCl in the presence of 0.125 to 1 μM of HBD2 or solvent control in final assay volumes of 1 mL in 12-well microtiter plate (Costar^®^ not treated, Corning). After 18 h incubation, the well contents were resuspended, transferred into microfuge tubes, and centrifuged at 5,000 × *g* for 10 min at 4°C to pellet the bacterial cells. Cells were then resuspended in 80 μL of 0.2 M Tris–HCl, pH 8.0. Then, 120 μL lysis buffer was added to the resuspended cells (final conditions were 200 μg/mL hen egg white lysozyme (Sigma-Aldrich), 20 mM sucrose and 0.2 mM EDTA in 0.2 M Tris-HCl, pH 8.0). After a 10 min incubation at RT, 2 μL of Protease Inhibitor Cocktail (Sigma Aldrich P8340) was added followed by 202 μL of extraction buffer (10 μg/mL DNAse I [Sigma-Aldrich DN25] in 50 mM Tris–HCl/10 mM MgCl_2_/2% Triton X-100). After 1.5 h incubation on a rocker at 4°C, samples were centrifuged at 1500 × *g* at 4°C for 5 min. The resulting supernatants from triplicate samples, which contain the outer membranes, were pooled and placed into 4 mL ultrafiltration tubes with 5 kDa cut off molecular weight (Amicon Ultracel, 5k, Millipore). PBS was added to yield a volume of 4 mL, and then the tubes were centrifuged at 2400 × *g* until about 500 μL residual volume was obtained. The outer membranes in this residual were then washed by suspending in 3.5 mL PBS and then centrifuging at 2400 × *g* for 25 min at RT, yielding a residual volume of approximately 200 μL. Of this, 4 μL were subjected to standard SDS-PAGE using Bio-Rad 16.5% Mini-Protean Tris-Tricine gels followed by silver stain. Images were acquired with Versadoc (Bio-Rad) and analyzed with Image Lab version 6.01 software from Bio-Rad Laboratories.

### Atomic Force Microscopy

*P. aeruginosa* (1 × 10^6^ CFU/mL inoculum) was incubated in 10% Mueller Hinton broth/140 mM NaCl/12.5 mM sodium phosphate pH 7.0 with and without HBD2 (0.25 μM), on glass coverslips (Borosilicate glass square coverslips, Thermo Fisher Scientific) in 6-well plates (Corning) for 18 h at 37°C. As negative controls for HBD2 the peptide solvent 0.01% acetic acid was included, respectively. Coverslips were then transferred into wells of a fresh six-well plate and adherent bacteria were fixed with 2.5% glutaraldehyde (Ted Pella, CA; 25%, electron microscopy grade) diluted in PBS for 20 min at 4°C followed by washing with deionized water according to Chao and Zhang, 2011 (64), and stored at 4°C until imaging by atomic force microscopy (AFM).

All AFM tests (65) were carried out with a NX12 AFM system (Park System) using an aluminum coated PPP NCHR (Park systems) cantilever with a spring constant of 42 N/m, a resonance frequency of 330 kHz, and a nominal tip radius of <10 nm. At least five images were acquired per sample in air with non-contact mode (NCM) with settings of 256 pixels/line and 0.75 Hz scan rate and continuous monitoring of the tip integrity. The images were first order flattened and the roughness and height of all bacteria were measured using XEI software (Park Systems). Specifically, roughness of each bacterium was calculated from the root mean square value (RMS, i.e., standard deviation of the distribution of height over the whole bacterium surface).

### Data and Statistical Analysis

Data graphs were generated using Microsoft Excel^®^ 2016 or GraphPad Prism 7.04 Software. Statistical analyses were performed using IBM SPSS version 24 or GraphPad Prism 7.04 Software. A *p*-value < 0.05 was considered statistically significant.

## Results

### At Low Concentrations, HBD2 Does Not Reduce Metabolic Activity but Inhibits Biofilm Production by *P. aeruginosa*, Unlike HBD3

**FIGURE 1:**
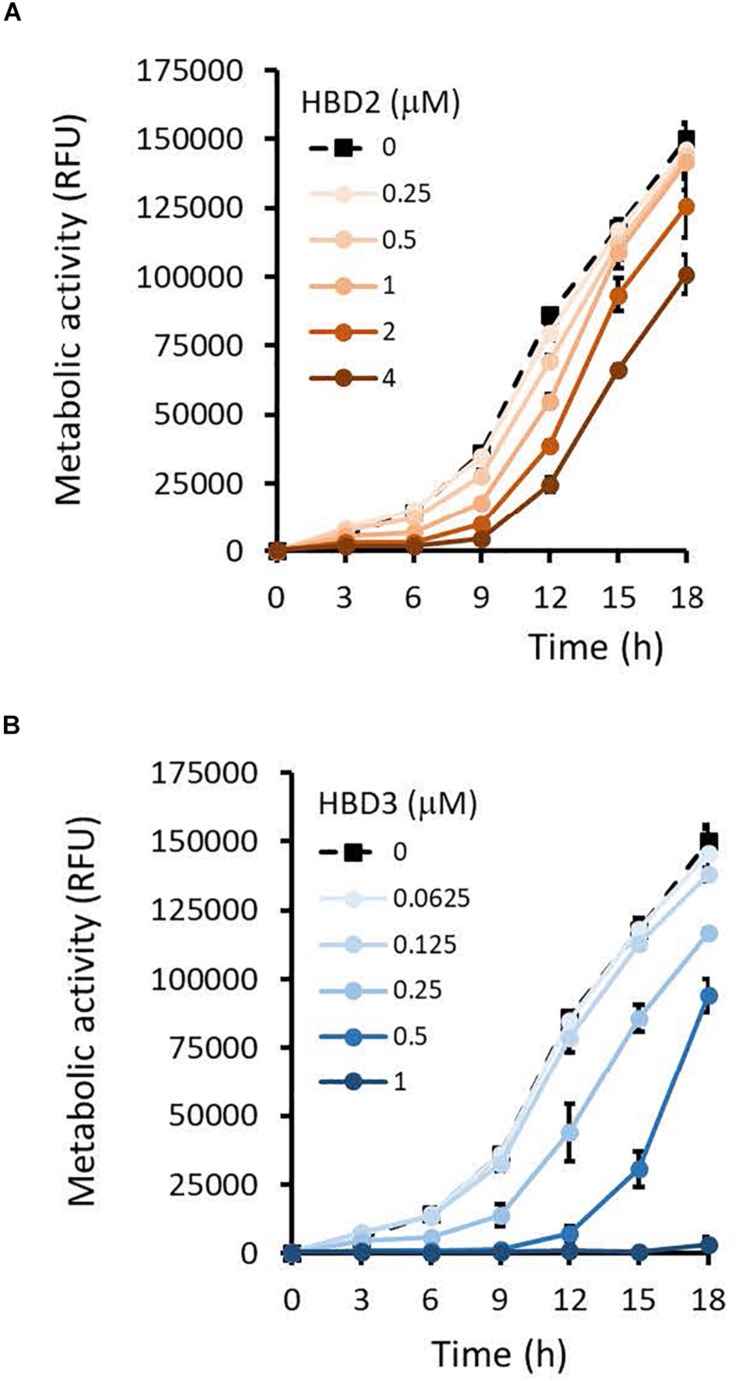
Metabolic activity of *P. aeruginosa* in the presence and absence of HBD2 and HBD3 over 18 h. Bacteria were incubated in 10% Mueller-Hinton/140 mM NaCl supplemented with 0.01% resazurin and fluorescence emitted by resorufin reflecting the production of reducing metabolites was measured every 3 h (530 nm_ex_, 616 nm_em_). Shown are the means ± SD of three independent experiments conducted in duplicates. RFU: relative fluorescence units. *p* < 0.001 for HBD2 **(A)** at 1, 2, and 4 μM and for HBD3 **(B)** at 0.25, 0.5, and 1 μM compared to the solvent control in univariate ANOVA with Bonferrroni *post hoc* analysis. All other concentrations were not significantly different from the solvent controls.

**FIGURE 2:**
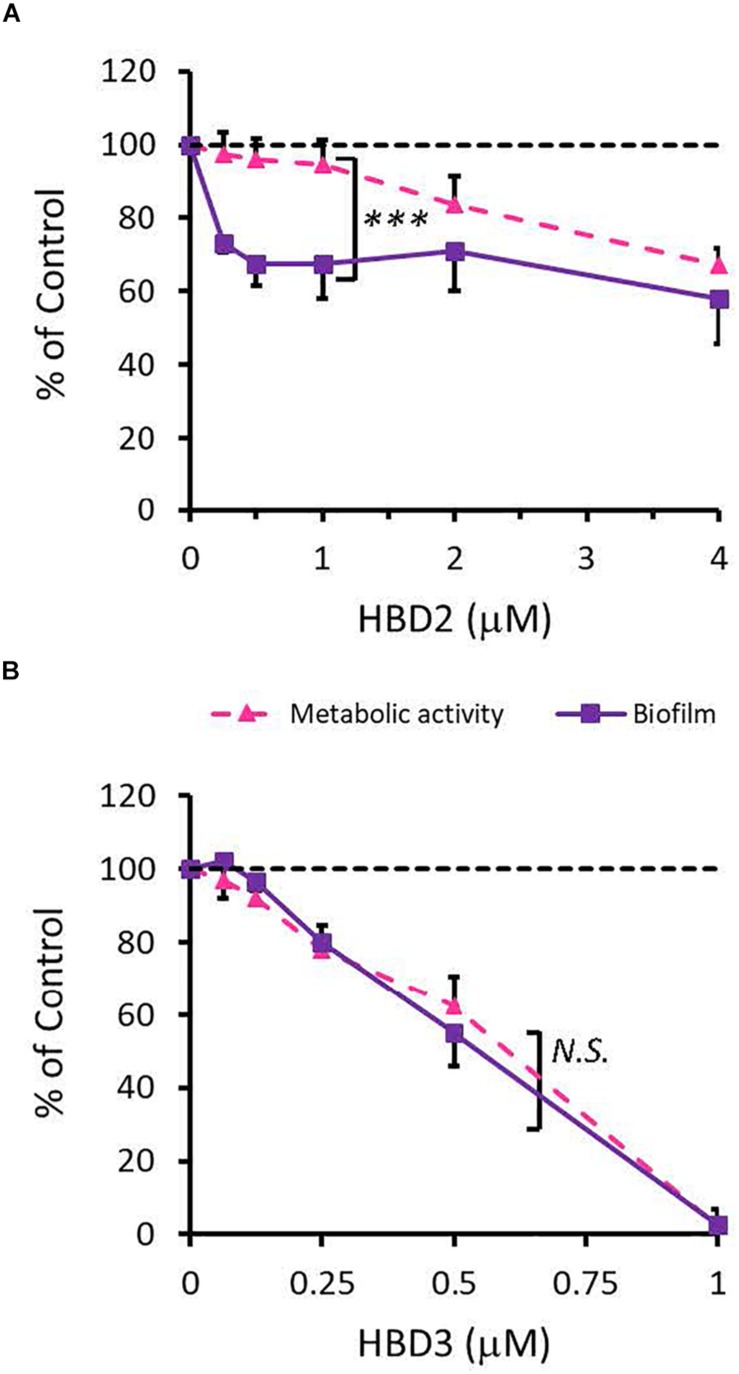
Comparative effects of HBD2 and HBD3 on *P. aeruginosa* biofilm and metabolic activity. Shown are biofilm formation and accumulated resorufin fluorescence after 18 h of incubation with HBD2 **(A)** and HBD3 **(B)** at the concentrations given. Data are expressed relative to the control and represent means ± SD of three independent experiments conducted in triplicates. ***p = 0.0004 in Two-way ANOVA. N.S: not significant (*p* = 0.7721).

**FIGURE 3:**
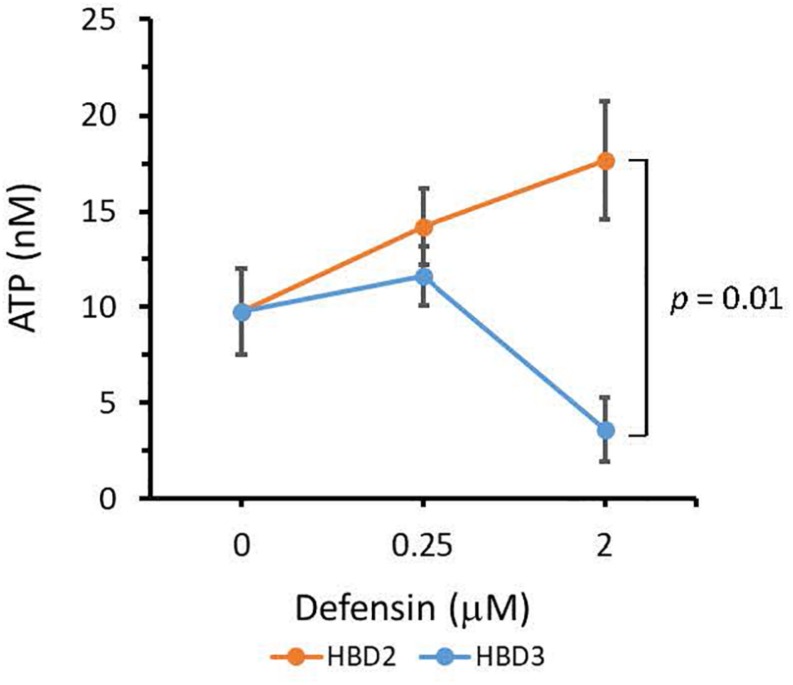
ATP quantification in *P. aeruginosa* after 18 h incubation in the presence or absence of HBD2 and HBD3 at the concentrations given. ATP concentrations are in nM and were calculated based on a standard curve. Shown are means ± SD of three independent experiments conducted in duplicates. *p* = 0.01 in One way ANOVA with Bonferroni *post hoc* analysis for 2 μM HBD2 compared to 2 μM HBD3.

To compare the antimicrobial activities of HBD2 and HBD3, *P. aeruginosa* was exposed to either peptide at various concentrations over a period of 18 h. Viability was assessed by measuring metabolic activity every 3 h via quantification of resazurin reduction to the highly fluorescent resorufin by bacterial metabolites. Biofilm was assessed at 18 h post-incubation via quantification of crystal violet staining through absorbance readings. The resazurin reduction assay showed that both HBD2 and HBD3 reduced metabolic activity in a dose-dependent manner, with HBD3 being more effective on a per molar basis, producing around a 30% reduction at 0.5 μM compared to the 4 μM needed by HBD2 at 18 h for the same effect (Figure 1). However, when comparing the effect on biofilm production between the two peptides, a notable difference was observed. At concentrations of 0.25 and 0.5 μM, HBD2 reduced *P. aeruginosa* biofilm to ∼75% of the control without significantly reducing the metabolic activity (Figure 2A). In contrast, at these concentrations, HBD3 reduced the formation of *P. aeruginosa* biofilm in a dose dependent manner that was directly proportional to the cumulative effect on metabolic activity and further reduced both biofilm and resorufin production to nearly undetectable levels at a concentration of 1 μM (Figure 2B) consistent with direct microbicidal activity. ATP concentrations measured at the end of the 18 h incubation period corroborated the resazurin data (Figure 3), showing maintained ATP levels in HBD2 treated bacteria but a significant reduction of ATP levels in HBD3 treated *P. aeruginosa* (at 2 μM defensin, 17.65 ± 5.31 nM ATP compared to 3.6 ± 2.88 nM ATP, respectively, *p* = 0.011). These data suggest a differential mechanism for the antimicrobial activity between HBD2 and HBD3, and that HBD2 selectively inhibits biofilm formation at low concentrations.

### HBD2 Similarly Inhibits Biofilm Production by *A. baumannii* Without Reducing Metabolic Activity at Lower Concentrations

**FIGURE 4:**
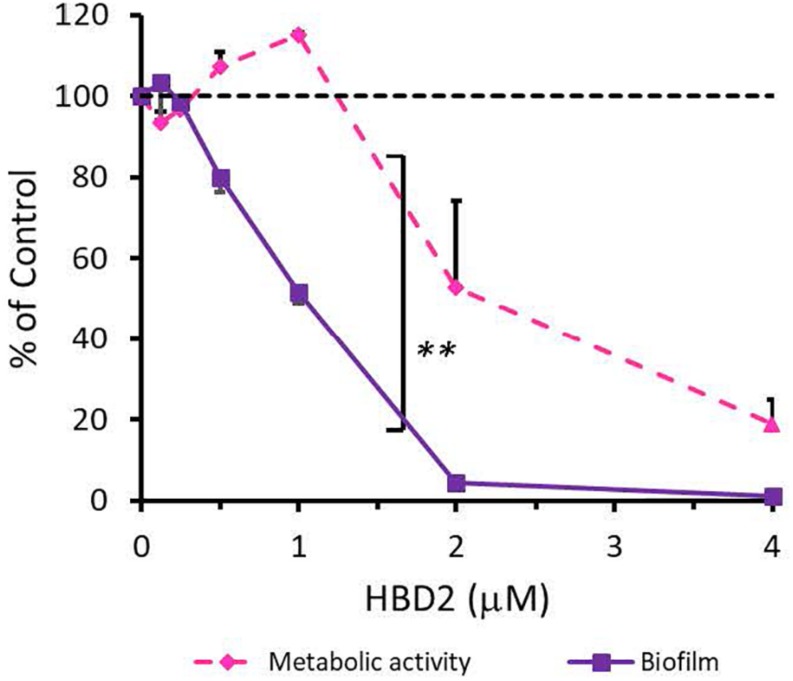
Effects of HBD2 on *A. baumannii* biofilm formation and metabolic activity. Shown are crystal violet absorbance and accumulated resorufin fluorescence expressed as % of the control after 18 h of incubation with HBD2 at the concentrations given. Data represent means ± SD of three independent experiments conducted in triplicates. **p = 0.004 for biofilm reduction versus reduction of metabolic activity in two tailed Paired Samples Test. In Oneway ANOVA with Bonferroni *post hoc* analysis, *p* = 0.001 for resazurin reduction at 4 μM HBD2, and *p* < 0.001 for biofilm reduction at 1, 2, and 4 μM HBD2, compared to the solvent control. All other data points were not significantly different from the control.

To rule out that the observed differential biofilm reducing activity of HBD2 activity was strain-specific and restricted to *P. aeruginosa*, we also subjected *A. baumannii*-another opportunistic Gram-negative rod of clinical relevance – to varying doses of HBD2 and determined resazurin reduction and biofilm production after 18 h incubation. As shown in Figure 4, at low concentrations, HBD2 similarly inhibited biofilm formation while not reducing metabolic activity of *A. baumannii*. For example, at 1 μM, HBD2 effected a significant reduction of biofilm to 51.77 ± 2.93% of the control (*p* < 0.001) while resazurin reduction was still at 115 ± 0.67% (*p* = 1.0) of the control (means ± SD, *n* = 3). At higher concentrations though, HBD2 appeared to have greater effects on *A. baumannii* compared to *P. aeruginosa* as both biofilm and metabolic activity were reduced to less than 2 and 20% of the control at 4 μM HBD2, respectively (1.23 ± 0.48 and 18.71 ± 10.43%, means ± SD, *n* = 3).

### HBD2 Biofilm Inhibitory Activity Does Not Depend on Chirality but on Folding State

**FIGURE 5:**
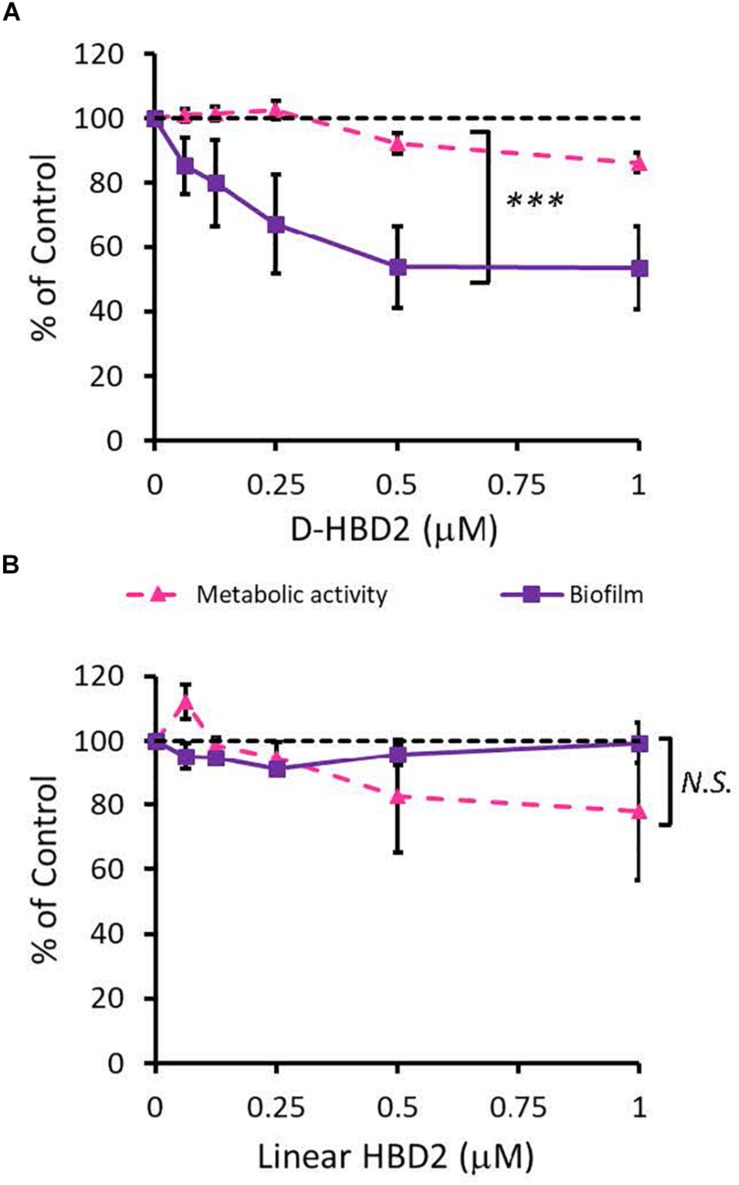
Comparative effects of D-HBD2 and linear HBD2 on *P. aeruginosa* biofilm and metabolic activity. Shown are biofilm formation and accumulated resorufin fluorescence expressed as % of the control after 18 h of incubation with all D-HBD2 **(A)** and linear HBD2 **(B)** at the concentrations given. Data represent means ± SD of three independent experiments conducted in triplicates. In paired *T* test comparing biofilm reduction and reduction of metabolic activity, ***p < 0.001 for D-HBD2 **(A)** and not significant (N.S.) for linear HBD2 **(B)**. In Oneway ANOVA with Bonferroni *post hoc* analysis, biofilm formation (*p* = 0.033) but not metabolic activity (*p* = 0.473) is significantly reduced by D-HBD2. For linear HBD2, none of the data is significantly different from the solvent control.

Since HBD2 appeared to selectively reduce biofilm formation and it has been known to bind to chemokine receptors on eukaryotic cells (66, 67), it was possible that the effects of HBD2 were due to binding to receptors involved in the biofilm regulatory pathway such as the GacA/GacS system. To test this, we assessed the activity of the D-form of HBD2, which, due to mismatched chirality, does not bind to proteinaceous receptors of L-HBD2. Like L-HBD2, D-HBD2 effected a significant reduction of biofilm production by *P. aeruginosa* without reducing metabolic activity (Figure 5A). Thus, this suggests that the observed HBD2 effect on *P. aeruginosa* biofilm production was not due to binding to receptors important for biofilm regulatory pathways.

Upon proper folding, defensins form three intramolecular disulfide bridges, which stabilize an amphipathic structure where cationic and hydrophobic amino acid residues are spatially segregated. To assess the importance of the structure and thus, charge distribution of HBD2 for its observed activity, a comparison was made between wildtype HBD2 and a linearized HBD2 mutant (Linear HBD2) with cysteine residues replaced by alanine residues. Loss of the cysteine residues prevents the formation of stabilizing disulfide bonds, drastically limits proper folding, and disrupts the organization of charged domains thought to be critical for AMP activity (68–70). As shown in Figure 5B, linearization of HBD2 resulted in a pronounced loss of activity.

Taken together, these data provided evidence for a receptor-independent activity that requires proper sequestration of charged and hydrophobic residues. We next asked whether HBD2 disrupts regulatory pathways of biofilm production through QS molecule binding. To answer this question, we took a three-pronged approach and performed *in silico* docking studies with known QS molecules involved in biofilm regulation, employed qPCR probing for genes differentially expressed during biofilm formation, and quantified pyorubin, a pigment regulated by the pathways that also affect biofilm production.

### HBD2 Binding to QS Molecules Is Unlikely Based on Autodock Vina Prediction

**FIGURE 6:**
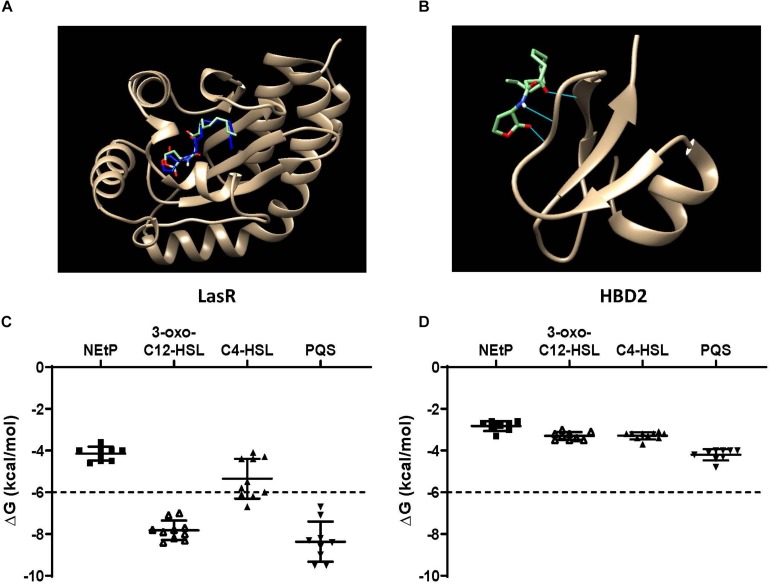
*In silico* docking and binding energies (ΔG) of various QS molecules calculated for LasR and HBD2. AutoDock Vina was used to predict binding sites and potential hits for HBD2 and quorum sensing molecules in comparison to LasR. **(A)** Test *N*-(3-oxohexanoyl) homoserine lactone (3-oxo-C12-HSL, green) lies inside the LasR binding pocket in the same region as co-crystallized 3-oxo-C12-HSL (blue) with LasR (RSCB 3IX3). **(B)** HBD2 does not contain a binding pocket for test 3-oxo-C12-HSL (green). Free energy of binding (ΔG) for various hits were determined for phosphorylcolamine (NEtP), 3-oxo-C12-HSL, *N*-butyryl homoserine lactone (C4-HSL), and 2-heptyl-3-hydroxy-4-quinolone (PQS) as ligands with either LasR **(C)** or HBD2 **(D)** as rigid receptors. Dashed lines indicate the –6 kcal/mol threshold for actively bound molecules.

QS molecules are small and flexible molecules with a potential for hydrogen bonding and hydrophobic interactions. Thus, they may bind to and be sequestered by HBD2. To explore this further, Autodock Vina was used (Figure 6) to predict HBD2 binding to known *P. aeruginosa* QS molecules representing three different QS systems, namely 3-oxo-C12-HSL – as the major QS molecule for *P. aeruginosa* utilized by the Las system, C4-HSL primarily utilized by the Rhl system, and PQS a key sensing molecule in the 4-quinolone system (71). As a positive control, Autodock Vina was also used to match the known binding pocket of the QS molecule 3-oxo-C12-HSL to its receptor LasR that has been previously assessed by X-ray diffraction (RSCB 3IX3) (72). Phosphorylcolamine (NEtP), which is not expected to bind to either LasR receptor or HBD2, was used as a negative control. Using the same methodology that confirmed binding of 3-oxo-C12-HSL to LasR here (Figure 6A) no binding of 3-oxo-C12-HSL to HBD2 was found (Figure 6B). Furthermore, we calculated the free energy of binding and found for LasR values corresponding to those reported in the literature (62, 73). Employing a −6 kcal/mol threshold for likely binding between ligand and receptor, binding between LasR and 3-oxo-C12-HSL, C4-HSL, and PQS was much more favorable (Figure 6C) than binding between HBD2 and these sensing molecules (Figure 6D).

Using Eq. (1), the dissociation constants (K_D_) for the most favorable binding pair between either LasR or HBD2 with each QS molecule was calculated (Table 3). This method predicted the K_D_ of 3-oxo-C12-HSL and LasR (1.15 μM) near that of previously reported values (∼5.5 μM) (74). Furthermore, K_D_ values for LasR binding with all three *P. aeruginosa* QS molecules were consistently two to three orders of magnitude lower than those of HBD2 binding with any of these QS molecules. This suggests that it is unlikely for HBD2 at physiological concentrations (75–77) to significantly bind these QS molecules.

**TABLE 3:** Dissociation constants for quorum sensing molecules calculated using AutoDock Vina measurements.

**Receptor**	**NEtP**	**3-oxo-C12-HSL**	**C4-HSL**	**PQS**
LasR	558 μM	1.15 μM	18.3 μM	191 nM
HBD2	4.637 mM	3.348 mM	2.417 mM	403 μM

*The best binding energies (predicted by AutoDock Vina) for each ligand-receptor pair were used to manually calculate dissociation constants (*K*_*D*_) using Eq. (1). NEtP: phosphorylcolamine; 3-oxo-C12-HSL: N-(3-oxododecanoyl) homoserine lactone; C4-HSL: N-butanoyl homoserine lactone; PQS: 2-heptyl-3-hydroxy-4-quinolone.*

### Gene Expression of *flgF* and *pslA* Is Not Affected by HBD2

**FIGURE 7:**
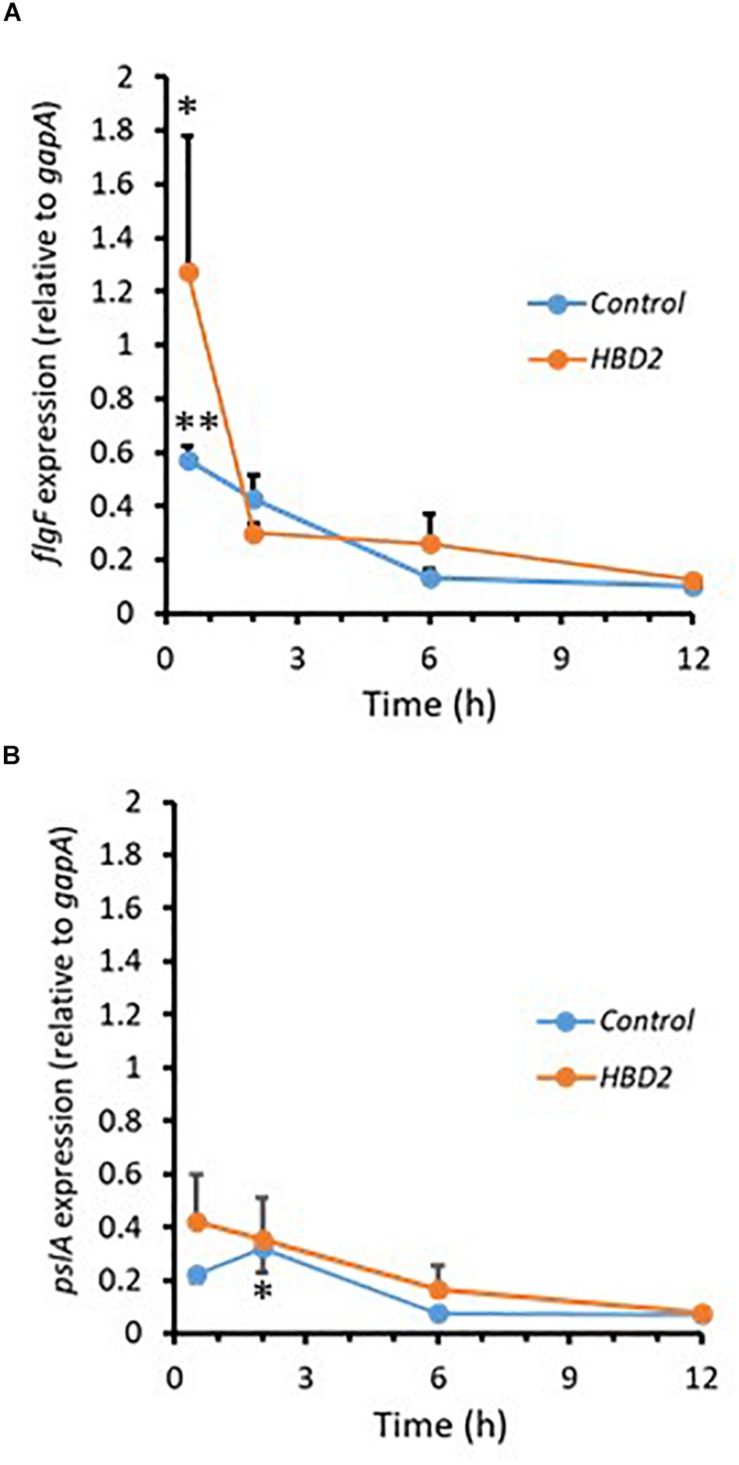
Relative gene expression of *flgF* and *pslA* in the presence and absence of 0.25 μM HBD2 as determined by qPCR. Gene expression of *flgF* **(A)** and *pslA* **(B)** in *P. aeruginosa* was calculated relative to the reference gene *gapA* after incubation in the presence or absence of HBD2 for up to 12 h. Shown are means ± SEM, *n* = 6. In multivariate ANOVA with Bonferroni *post hoc* analysis (*p < 0.05 and **p < 0.01), gene expression of *flgF* and *pslA* changed over time (Control: *p* < 0.01 for *flgF* 0.5 h versus 6 h and 12 h, and *p* < 0.05 for *pslA* 2 h versus 6 h and 12 h; HBD2: *p* < 0.05 for *flgF* 0.5 h versus 12 h) but there was no significant difference between the control and HBD2 treated bacteria.

During biofilm formation, motility and production of exopolysaccharide are reciprocally regulated with reduction of the expression of flagella-related genes and increase in the expression of genes contributing to polysaccharide synthesis including Psl polysaccharide. Thus, we compared the expression of *flgF* (Figure 7A) and *pslA* (Figure 7B) in *P. aeruginosa* treated with 0.25 μM HBD2 or solvent at various timepoints for up to 12 h. For solvent treated control bacteria, as expected, *flgF* gene expression decreased within 2 h reaching statistical significance after 6 h and the expression of *pslA* was signficantly increased after 2 h compared to the later time points (*p* < 0.01 and *p* < 0.05 in multivariate ANOVA with Bonferroni *post hoc* analysis). As observed for control bacteria, *flgF* gene expression decreased over time and was significantly reduced in HBD2 treated bacteria (*p* < 0.05) though changes in *pslA* gene expression did not reach statistical significance. However, there was overall no statistical significant difference between solvent and HBD2 treated bacteria. Thus, expression analysis of genes altered early in the biofilm production process does not support that HBD2 interference with biofilm production occurs at the transcriptional level.

### Pyorubin Accumulation Is Not Reduced in Media Collected From HBD2 Treated *P. aeruginosa*

Pigment production in *P. aeruginosa* has been shown to be also regulated by QS (24, 61). To further corroborate that HBD2 does not interfere with QS, we quantified pyorubin released into culture supernatants in the presence and absence of HBD2. At 0.125 and 0.25 μM HBD2 there was no difference in pyorubin accumulation compared to the control (data not shown). In the presence of 0.5 and 1 μM HBD2, there was a slight increase of pyorubin (109.5 ± 4.9 and 109.9 ± 5.8% of the control, respectively, *p* < 0.01 in univariate ANOVA with Bonferroni *post hoc* adjustment). This finding further supports that HBD2 does not inhibit quorum sensing and next, we explored whether HBD2 may induce structural changes in the outer membrane that could interfere with the transport of biofilm precursors to the extracellular space.

### HBD2 Alters the Outer Membrane Protein Profile of *P. aeruginosa*

**FIGURE 8:**
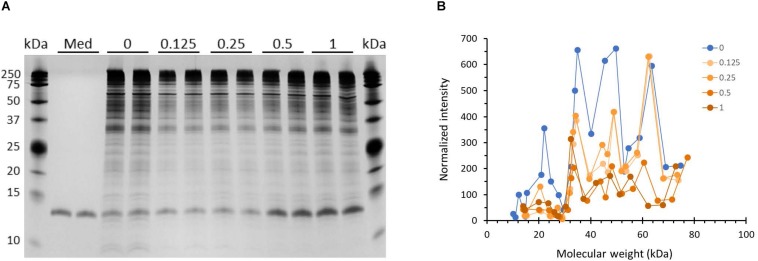
Outer membrane protein profile of *P. aeruginosa* after 18 h incubation in the presence and absence of HBD2. **(A)** Four μL of concentrated outer membrane preparations from HBD2 treated (0.125–1 μM) or solvent control exposed bacteria (0) were resolved by SDS Tris-Tricine PAGE and visualized by silver stain. (Med) indicates medium only processed like bacteria-containing samples. The band migrating between 10 and 15 kDa in all samples is consistent with the expected molecular weight of lysozyme (14 kDa) that was added to the extraction buffer. **(B)** Approximate molecular weight and intensities of bands were quantified with Image Lab software and band intensities detected in both replicates were normalized to the intensity of the presumptive lysozyme band. Each data point represents the average of replicates. Each line represents the protein profile for the indicated HBD2 concentration (in μM).

Outer membrane proteins participate in the process of biofilm formation (78). Hence, we probed whether incubation with HBD2 leads to changes in the outer membrane protein profile of *P. aeruginosa* (Figure 8). A representative image of outer membrane preparations resolved by silver stained SDS-PAGE is depicted in Figure 8A. Numerous bands are detected ranging from about 10 kDa to over 200 kDa with the most dominant bands appearing above 25 kDa, in particular a band around 35 kDa similar to the molecular weights of previously reported *P. aeruginosa* outer membrane proteins (79). Two weaker bands around 10 kDa are consistently visible only in the outer membrane preparations from control bacteria. Overall, the outer membranes from HBD2 treated bacteria appear to contain less proteins between 35 and 75 kDa. A prominent band between 10 and 15 kDa is detected in all samples, including the medium control, consistent with the molecular weight of the lysozyme (14 kDa) added during the extraction procedure. Figure 8B summarizes the protein profiles of the outer membrane preparations from control bacteria and HBD2 treated bacteria. To control for variations during the ultrafiltration process and gel loading, the band intensities of the various proteins were normalized with the presumptive lysozyme band intensity. HBD2 appears to affect a decrease in outer membrane proteins in particular at about 22, 34, 40, 45, and 50 kDa, with the changes noticeable at all concentrations tested.

### Atomic Force Microscopy Reveals Ultrastructural Changes in HBD2 Treated Bacteria Reflected in Increased Surface Roughness

**FIGURE 9:**
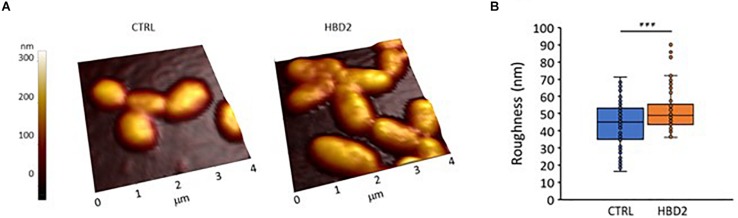
Atomic force microscopy of *P. aeruginosa* after 18 h incubation in the presence and absence of 0.25 μM HBD2. Bacteria were incubated on glass slides and fixed with 2.5% glutaraldehyde prior to imaging. Images taken with the atomic force microscope were first order flattened before extracting measurement for bacterial roughness. **(A)** Representative images. CTRL: solvent control exposed bacteria. **(B)** Box and whisker chart (with inner points and outliers) of roughness measurements from multiple images of solvent exposed control bacteria (CTRL, *n* = 85) and 0.25 μM HBD2 treated bacteria (*n* = 69). ***p < 0.001 in independent samples *T* test.

We also assessed whether the changes at the outer membrane induced by HBD2 resulted in topographical changes and employed atomic force microscopy to measure bacterial height and roughness after incubation for 18 h in the presence or absence of 0.25 μM HBD2 (Figure 9). Representative images of control and HBD2 treated bacteria are shown in Figure 9A. The surface of control bacteria appears smoother compared to the surface of HBD2 treated bacteria, the latter showing irregular dents. While the overall bacterial height is not significantly different in HBD2 treated bacteria compared to solvent only exposed bacteria (215.22 ± 3.96 nm versus 220.24 ± 3.23 nm, means ± SEM, *n* = 85 and *n* = 69, respectively, *p* = 0.343), there is a significant increase in roughness in HBD2 treated samples (Figure 9B) consistent with a structurally altered surface (43.39 ± 1.52 versus 51.86 ± 1.5 nm, means ± SEM, *n* = 85 and *n* = 69, *p* < 0.001 in independent samples *T* test).

Taken together, our data demonstrate that at low concentrations L- and D-forms of HBD2 inhibit biofilm formation while not reducing metabolic activity in Gram-negative bacteria of two different genera, *Pseudomonas* and *Acinetobacter*. Furthermore, this activity appears to be receptor-independent and not mediated by interference with quorum sensing or other regulatory pathways of biofilm production at the transcriptional level. Instead, our data are consistent with structural changes induced by HBD2 that interfere with the transport of biofilm precursors into the extracellular space suggesting a novel mechanism of action for the antimicrobial peptide HBD2.

## Discussion

In this study, we demonstrate that, HBD2, at nanomolar concentrations, and independent of its chiral state, significantly reduced biofilm formation of *P. aeruginosa* without affecting metabolic activity. This was unlike HBD3, which equally reduced biofilm and metabolic activity at nanomolar concentrations. HBD2 similarly affected *A. baumannii*, another Gram-negative bacterium, at low concentrations. *In silico* modeling did not support direct binding of HBD2 to QS molecules, the release of a QS regulated pigment was not inhibited, and the expression of biofilm-related genes was not influenced by HBD2. However, the outer membrane protein profile was altered in HBD2 treated bacteria with reduced representation of several proteins, which was accompanied by increased roughness of the bacterial surface. Taken together, these data support a novel mechanism of biofilm inhibition by HBD2 at low concentrations that is independent of biofilm regulatory pathways but involves structural changes induced by HBD2 that may interfere with the transport of biofilm precursors into the extracellular space.

HBD2 has been previously reported to reduce bacterial survival in existing biofilm cultures of *Lactobacillus* ssp., Gram-positive bacteria, at higher, micromolar concentrations (80). However, inhibition of biofilm formation by HBD2 has not been reported previously to the best of our knowledge. Considering the rapid induction of HBD2 in epithelial cells’ response to proinflammatory cytokines and bacterial challenge (81), the ability to interfere with biofilm formation at low concentrations adds importance to the role of HBD2 in innate host defense during the early interaction between host and pathogen. Bacteria are more susceptible to host-derived and exogenous antimicrobial agents while they are metabolically active in the planktonic state prior to biofilm production. Thus, HBD2 may amplify host defenses early in the attempted infection process and could improve the action of antibiotics in a clinical setting (82). Synergism studies will be able to address this experimentally in the future.

Anti-biofilm activity of HBD2 in the absence of inhibition of metabolic activity of *P. aeruginosa* occurred only at low concentrations. A concentration dependent mechanism of action has been well documented for the lantibiotic nisin, which, at nanomolar concentrations, preferentially binds to lipid II disrupting cell wall synthesis and, at micromolar concentrations, embeds into the bacterial membrane causing pore formation (83–87). More recently, the alpha-defensin human neutrophil peptide 1 (HNP1) has been added to the list of AMPs that initially interact with lipid II, and when concentrations increase, with the bacterial cell membrane (88). Binding of HBD3 to lipid II has also been described, albeit at higher concentrations, in the micromolar range (47). It is conceivable that HBD2 could similarly interfere with membrane-embedded proteins responsible for the transport of biofilm components (17) at low concentrations followed by membrane perturbation at higher concentrations.

The differential effect of HBD2 on biofilm production and metabolic activity of *P. aeruginosa* was not observed in the related beta-defensin HBD3, which was active at lower concentrations than HBD2 and equally reduced biofilm and metabolic activity reflecting a strong bactericidal activity. These differences in their activity could be at least in part attributed to the differences in their physicochemical properties with respect to net charge, surface charge distribution, hydrophobicity index, and behavior in solution (51, 89). Biofilm is a complex matrix with numerous components that can be affected in different ways by HBD2 and HBD3. For example, alginate has been shown to affect antimicrobial peptide conformation inducing alpha-helices contigent on the hydrophobicity (90), and HBD2 and HBD3 substantially differ in their hydrophobicity with HBD2 being more hydrophobic than HBD3.

HBD2, at low concentrations, similarly inhibited biofilm production in *A. baumannii* without reducing metabolic activity suggesting the observed effects are not strain specific. However, at higher HBD2 concentrations differences between the effects on *P. aeruginosa* and *A. baumannii* emerged as reflected in a near complete inhibition of biofilm production of *A. baumannii* contrasting the stalled biofilm inhibition of *P. aeruginosa*. The lesser susceptibility of *P. aeruginosa* to HBD2 may be due to a greater outer membrane vesicle production in *P. aeruginosa* that may sequester HBD2 before it reaches the bacterial cell (91).

Like other defensins, HBD2 forms three intramolecular disulfide bridges and linearization of the peptide can reveal the importance of its structure for its antimicrobial activity (92). Here, linearization of full length HBD2 led to a pronounced loss of both its antimicrobial and biofilm inhibitory activity. This contrasts reports for other defensins including HBD3 and could be attributed to a lack of accumulation of positively charged amino acid residues at the C-terminus of HBD2 compared to HBD3. Chandrababu et al. (93) have shown that positively charged residues cluster in the C-terminal segment of a linearized form of HBD3 allowing them to interact with the negatively charged phospholipids of micelles. The inherent antimicrobial activity of this patch of cationic residues is also reflected in studies with HBD3 analogs truncated to the C-terminal region (94). The here observed loss of activity after linearization could indicate that HBD2 functions through a receptor (56). However, D- and L forms of HBD2 did not differ in their activity and thus, we interrogated the possibility that HBD2 interferes with regulatory networks of biofilm production.

QS molecules are key to the regulation of virulence factor production including biofilm and pigment in *P. aeruginosa*. They are small hydrophobic molecules (95) and thus, we interrogated possible binding of HBD2 to QS molecules *in silico*. We found favorable binding of LasR to not only its cognate ligand 3-oxo-C12-HSL but also to C4-HSL and PQS. This is in line with a recent study describing LasR as promiscuis for binding a variety of QS molecules (96). The unfavorable binding energies derived for HBD2 suggest that interference of QS-dependent processes through direct HBD2 binding to individual QS molecules is unlikely. Another type of QS molecule, (2*S*,4*S*)-2-methyl-2,3,3,4-tetrahydroxytetrahydrofuran-borate (S-THMF-borate), has been shown to increase biofilm formation in *P. aeruginosa* (97, 98). However, although S-THMF-borate – a molecule with a distinct structure from major Gram-negative QS molecules – has been identified in some Gram-positive and Gram-negative bacteria (99), *P. aeruginosa* does not encode the *luxS* gene required for its synthesis (100) and binding to this S-THMF-borate should not be further considered as an underlying mechanism for the observed biofilm inhibition.

In agreement with the *in silico* data, HBD2 did not affect the expression of *flgF* and *pslA*. Thus, interference of HBD2 with regulatory networks at the transcriptional level is not likely to account for its biofilm inhibitory activity. However, we cannot rule out that HBD2 has posttranscriptional effects through interference with the two component signal transduction system GacS/GacA (71, 101). GacS is a transmembrane sensor kinase phosphorylating GacA, which in turn induces the expression of small RNA molecules that antagonize the protein RsmA, a translational repressor interfering with *psl* translation and known to normally block exopolysaccharide production (102). It is conceivable that HBD2 could interfere with GacS upon inserting into the bacterial membrane. Finally, HBD2 might bind to the secondary messenger molecule c-di-GMP, which regulates biofilm formation in *P. aeruginosa* at multiple levels (103). Previously, de la Fuente-Nunez et al. (104) demonstrated that peptide 1018, derived from the antimicrobial peptide bovine Bac2a (105), inhibited biofilm formation in *P. aeruginosa* while not affecting planktonic growth by binding to the second messenger p(pp)Gpp and promoting its degradation. A similar mode of action could apply to HBD2.

Further supporting that HBD2 does not act through interference with regulatory networks is our finding that pyorubin accumulation in the extracellular fluid was not diminished after incubation with HBD2. Pyorubin is composed of several pigments including aeruginosin A, which is a phenazine, like the much better studied *P. aeruginosa* pigment pyocyanin (106). Phenazines typically traverse the bacterial membrane freely and their production is under the same controls that govern biofilm production (107, 108).

Considering the lack of evidence for interference with regulatory networks and the stereoisometry independent activity of HBD2, we conceived that the observed HBD2 mediated inhibition of biofilm production is most likely due to embedding in the bacterial membrane and disruption of transport of biofilm precursor molecules across the membrane. An increasing amount of research suggests that AMPs can target discrete loci in bacterial membranes and thereby disrupt biological processes (109). For example, AMPs are known to impair the assembly of multicomponent enzyme complexes in the bacterial cell membrane (110) or disrupt periplasmic protein-protein interaction interfering with molecular transport (111). In 2013, Kandaswamy et al. showed that HBD2 localizes to the mid-cell region of the Gram-positive bacterium *E. faecalis* (112). The authors determined that this mid-cell region is rich in anionic phospholipids and that HBD2 delocalized the spatial organization of protein translocase SecA and sortases, both of which are important for pilus biogenesis (112, 113). It is possible that HBD2 targets similar machinery in *P. aeruginosa* to impair biofilm formation. SecA also plays a role in the transport of outer membrane proteins in Gram-negative bacteria (114) and outer membrane proteins have been shown to participate in biofilm formation, including the 11 kDa LecB protein and the 38 kDa OprF (115, 116). Consistent with this we found an altered outer membrane protein profile in HBD2 treated bacteria with a paucity of proteins around 10 kDa and proteins around the molecular weights of previously reported outer membrane proteins. This may indicate structural changes of the outer membrane, which was further supported by our atomic force microscopy data demonstrating an increased roughness of the bacterial surface after HBD2 treatment. It is important to note, however, that increased roughness could also represent changes in the LPS profile. Atomic force microscopy has been previously employed elsewhere to demonstrate outer membrane damages in *P. aeruginosa* (117). Resolving the outer membrane proteins by 2D gel electrophoresis could further delineate the observed changes in future experiments, which should also revisit the action of the D-form of HBD2 and effects on the outer membrane of *A. baumannii*. Finally, outer membrane vesicles have been recognized to take part in the formation of biofilm by interacting with extracellular DNA and HBD2 interference with proper outer membrane formation may disrupt this process (118).

## Conclusion

This study reveals distinct activity of two epithelial beta-defensins, HBD2 and HBD3, and provides evidence for a novel antibacterial action of HBD2. At low concentrations in the nanomolar range, HBD2 reduced biofilm formation without reducing the metabolic activity of *P. aeruginosa*. Biofilm production of *A. baumannii* was similarly affected, indicating that the observed HBD2 activity is not strain specific. This activity is unlikely mediated through a receptor-dependent interference with regulatory networks but contingent on preservation of HBD2 structure. Our findings are consistent with a membrane-targeted action of HBD2 that affects proper function of membrane-associated proteins involved in biofilm precursor transport into the extracellular environment. Future studies dissecting the molecular basis for the described HBD2 activity may inform the development of new methods for the manipulation of biofilms in aquaculture, in the food industry, and in the healthcare setting, which is in particular of interest for the latter considering the rise of multidrug resistance.

## Data Availability Statement

The raw data supporting the conclusions of this article will be made available by the authors, without undue reservation, to any qualified researcher.

## Author Contributions

KP, BB, TY, MB, EE, AT, AC, and YW: acquisition of the data. KP, BB, TY, MB, EE, AT, AC, HP, YW, WL, and EP: analysis and interpretation of the data. KP, MM, and CA: method development. KP and EP: statistical analysis. KP: molecular docking. KP, HP, YW, WL, and EP: conceptual and experimental design. KP, HP, and EP: drafted the manuscript. KP, BB, TY, MB, EE, AT, AC, MM, CA, HP, YW, WL, and EP: critical revision of the manuscript for important intellectual content. All authors approved the final manuscript submission.

## Conflict of Interest

The authors declare that the research was conducted in the absence of any commercial or financial relationships that could be construed as a potential conflict of interest. The handling Editor declared a past co-authorship with one of the authors WL.

## Footnotes

1 https://www.cgl.ucsf.edu/chimera/
